# Impact of the SARS-CoV-2 infection in individuals with sickle cell disease: an integrative review

**DOI:** 10.3389/fmed.2023.1144226

**Published:** 2023-05-02

**Authors:** Laura Resende Guimarães Pereira, Maria Vitoria Gomes da Silva, Carla Maria Ramos Germano, Isabeth F. Estevao, Débora Gusmão Melo

**Affiliations:** Department of Medicine, Federal University of São Carlos (UFSCar), São Carlos, Brazil

**Keywords:** sickle cell disease, sickle cell trait, COVID-19, SARS-CoV-2 infection, acute chest syndrome, risk factors, hydroxyurea, disease management

## Abstract

Sickle cell disease is the most common hemoglobinopathy among humans. As the condition promotes susceptibility to infections, chronic inflammation, and hypercoagulability disorders, several international agencies have included individuals with this disease in the COVID-19 risk group for severe outcomes. However, available information about the subject is not properly systematized yet. This review aimed to understand and summarize the scientific knowledge about the impact of SARS-CoV-2 infection in patients with sickle cell disease. Searches were performed in the Medline, PubMed, and Virtual Health Library databases based on descriptors chosen according to the Medical Subject Headings. We analyzed studies published between 2020 and October 2022, developed with qualitative, quantitative, or mixed methodology, and written in English, Spanish, or Portuguese. The search resulted in 90 articles organized into six categories. There is disagreement in the literature about how different aspects related to sickle cell disease, such as chronic inflammation status, hypercoagulability, hemolytic anemia, use of hydroxyurea, and access to medical care interference with the clinical course of COVID-19. These topics deserve further investigation. It is evident, however, that the infection may manifest in an atypical way and act as a trigger for the development of sickle cell-specific complications, such as acute chest syndrome and vaso-occlusive crises, conditions that are associated with great morbidity and mortality. Therefore, healthcare professionals must be aware of the different forms of presentation of COVID-19 among these individuals. Specific guidelines and therapeutic protocols, as well as public policies for sickle cell individuals, must be considered.

**Systematic review registration:**

This review (https://doi.org/10.17605/OSF.IO/NH4AS) and the review protocol (https://osf.io/3y649/) are registered in the Open Science Framework platform.

## 1. Introduction

Sickle cell disease is the most frequent hemoglobinopathy in humans (1). At least 300, 000 children are born with the condition each year (2, 3). It is an inherited hemolytic anemia caused by a genotype that determines the production of type S hemoglobin (HbS), a variant of adult hemoglobin (HbA), as the result of a single mutation (missense mutation) in the β-globin gene, which consists of a thymine nucleotide substitution for adenine. This event changes the codon of the sixth amino acid in the β-globin chain from glutamic acid to valine (3).

The homozygous form of the HbS gene characterizes sickle cell anemia, the most prevalent monogenic condition in the world. However, other genotypes causing sickle cell disease can be identified. Basically, they correspond to the uniparental inheritance of the βS allele in concomitance with mutations for other HbA variants, such as S/β°thalassemia, S/β+ thalassemia, and SC (1, 4). Although there may be some variation in the severity of the clinical phenotype for each of the genotypes associated with sickle cell disease, the pathophysiology of this entity is multisystemic and, regardless of the origin genotype, is fundamentally anchored in HbS polymerization, vaso-occlusion, and hemolytic anemia (3). A carrier of sickle cell, also known as having the sickle cell trait, is an individual who, despite being heterozygous for the HbS gene, can synthesize enough HbA to inhibit polymerization and, generally, does not exhibit relevant clinical manifestations (4).

It is possible to group the most relevant chronic complications of sickle cell disease into two categories: those related to large vessel vasculopathy, such as cerebrovascular disease, pulmonary hypertension, priapism, and retinopathy; and those that contribute to progressive ischemic organ damage, such as hyposplenia, renal failure, bone disease, and liver injury. Damage to the spleen, which can culminate in functional asplenia, confers an increased risk of infections, representing an additional factor in higher mortality, especially in children. As for acute complications, pain crises and acute chest syndrome, one of the main causes of death in this population, are the most frequent manifestations. Despite advances in medical care, the life expectancy of individuals with sickle cell disease is still reduced by about two to three decades compared to the general population. Child mortality in this group can reach 90% in low-income countries (2, 3, 5, 6).

In light of the high prevalence of sickle cell disease in different populations and the well-known susceptibility of these individuals to infections (2, 7), it is essential to understand the pathophysiology of COVID-19 in the context of sickle cell disease. The World Health Organization has recorded over 6 million deaths worldwide due to COVID-19 (8). Furthermore, the statistics reveal that the elderly, especially those over 60 years old, and people with underlying chronic diseases, such as hypertension, diabetes, cardiovascular disease, chronic kidney disease, cancer, obesity, and chronic obstructive pulmonary disease, are more susceptible to the severe forms of the disease, with increased mortality rates (9–12). Although several specialized agencies around the world, such as the Centers for Disease Control and Prevention (CDC), have included people with sickle cell disease as part of the risk group for poor outcomes of COVID-19 (13), the available information on the impact of infection on these individuals has not yet been properly systematized.

It seems that the clinical course of COVID-19 in this population is quite variable. Some studies point out, for example, that SARS-CoV-2 infection may act as a trigger for the occurrence of typical complications of sickle cell disease, in particular vaso-occlusive crises and acute chest syndrome, but the data on mortality and hospitalizations, in principle, do not exhibit significant differences compared with the general population (14–16).

Therefore, this review aimed to provide a detailed overview of the scientific production on sickle cell disease and COVID-19 in order to understand and systematize the knowledge produced on the subject. Ultimately, it intends to offer subsidies to guide decision-making in clinical practice, the formulation of guidelines and therapeutic protocols, and the development of public policies directed at this population.

## 2. Methods

This is an integrative review conducted according to the literature (17–19), based on PRISMA recommendations (20), and with a research protocol registered in the *Open Science Framework* (https://osf.io/3y649/). The intent of this review was to answer the following research question: “What is the impact of SARS-CoV-2 virus infection in people with sickle cell disease?” outlined in the PICO format (21, 22), as detailed in Table 1.

**Table 1:** Research question definition by following PICO parameters.

**P**	**Population**	**Who was studied?**	**People with sickle cell disease**
**I**	Interest/Intervention	What happened?	Infection caused by the SARS-CoV-2 virus
**C**	Comparison	Comparison between different populations	People without sickle cell disease
**O**	Outcomes	What are the clinical features of SARS-CoV-2 infection? What is the prognosis?	Natural history of the disease, prognosis, mortality and lethality of SARS-CoV-2 infection in people with sickle cell disease.

### 2.1. Search strategy

The searches were performed in three databases: Virtual Health Library (Biblioteca Virtual da Saúde, BVS), PubMed, and Web of Science. Using the BVS, we were able to access the LILACS (Latin American and Caribbean Literature on Health Sciences) database, which includes SciELO (Scientific Electronic Library Online), medRxiv, bioRxiv, and ColecionaSUS. In PubMed, we used the MEDLINE database. The Web of Science was searched by topics and all collections were accessed.

The following descriptors, extracted from the “Medical Subject Headings” platform (https://www.nlm.nih.gov/mesh/), were used in the search: [(HbS Disease) OR (Hemoglobin S Disease) OR (Sickle Cell Anemia) OR (Sickle Cell Disease) OR (Sickle Cell Disease) OR (Sickle Cell Disease due to Hemoglobin S)] AND [(SARS-CoV-2) OR (SARS-CoV-2 Virus) OR (SARS-CoV-2 Infection) OR (COVID- Virus 19) OR (COVID-19 Virus) OR (Pandemic COVID-19) OR (Pandemic COVID-19) OR (Pandemic COVID-19) OR (COVID-19 Virus) OR (COVID-19 Virus Disease) OR (COVID-19 Virus Infection) OR (2019 Novel Coronavirus) OR (2019 Novel Coronavirus Disease) OR (2019 Novel Coronavirus Infection) OR (2019-nCoV) OR (2019-nCoV Disease) OR (2019-Infection nCoV) OR (2019 Coronavirus Disease) OR (2019 Coronavirus Disease) OR (2019 Coronavirus Virus) OR (2019 Coronavirus Infection) OR (SARS Coronavirus 2) OR (SARS Coronavirus 2)] AND [(inflammation) OR (mediators of inflammation) OR (innate inflammatory response) OR (embolism and thrombosis) OR (thrombosis) OR (blood clot) OR (thrombus) OR (hypercoagulability) OR (thromboembolism) OR (physiology) OR (oxidative stress) OR (oxidative injury) OR (hemolysis)].

The research was conducted independently by two researchers with the help of a third collaborator, who was consulted to resolve any doubts. The concordance between the searches in the three databases was checked in terms of the date and quantitative characteristics of the data collected. After confirming the compatibility of the information, the identified publications were entered into the Rayyan software (https://www.rayyan.ai/), a tool specifically developed to assist in the primary selection of articles in systematic review projects (23).

Using Rayyan, duplicates were identified and excluded. Then, an initial analysis of the papers was performed by reading the title, abstract, and keywords. Articles that were not aligned with the research question were eliminated. In this first screening, the reading and selection of the documents were conducted by two researchers in a “blind” mode to avoid bias. Doubts and disagreements were discussed, solved together, and then, if necessary, triple-checked with the other researcher to reach a final decision.

The articles that remained under analysis were entirely read and submitted to a new selection stage after filling out an eligibility form (Form 1-Supplementary material) based on the inclusion and exclusion criteria. Additionally, the references of these articles were reviewed in a manual search for other manuscripts pertinent to the study. In this step, the selection was performed with the help of the Mendeley reference manager (https://www.mendeley.com/) (24).

### 2.2. Inclusion and exclusion criteria

This review included theoretical or empirical studies, primary or secondary research, carried out with qualitative, quantitative, or mixed methodology, written in Portuguese, English, or Spanish, that portray the theme of interest and were published between January 2020 and the day of the last database search, performed on October 7, 2022.

### 2.3. Assessment of the level of evidence of the studies and data extraction

Selected papers were classified according to their levels of evidence, taking into consideration the type of study, according to criteria adapted from Melnyk and Fineout- Overholt (25) (Table 2). Their data were systematically extracted using a standardized form (Form 2-Supplementary material) that includes information about authorship, country of origin, type of study, year of publication, main outcomes, and conclusions, allowing the synthesis of the information found and the evaluation of the consistency and validity of the studies.

**Table 2:** Categorization of the level of evidence, according to the type of study of the scientific productions.

**Type of evidence**	**Level of evidence**	**Description**
Systematic review and meta-analysis	I	Systematic review meta-analysis- of all randomized clinical trials
Randomized controlled trial	II	Well-designed, randomized, controlled clinical trial.
Controlled trial without randomization	III	Well-designed non-randomized controlled clinical trial.
Case-control or cohort study	IV	Well-designed case-control or cohort clinical study.
Systematic review of qualitative or descriptive studies	V	Systematic review of qualitative, descriptive studies – for example, systematic review of case series, qualitative research.
Qualitative or descriptive stud	VI	Single descriptive or qualitative study – for example, ecological studies, cross-sectional studies, case reports, case series.
Expert opinion or consensus	VII	Opinion study, editorial, narrative review.

Adapted from Stillwell et al. (26).

Once the data were extracted, the results were compared and problematized. The objective was to integrate the knowledge through a synthesis of the findings. Therefore, the similarities of the studies were explored by placing them into categories to organize the data clearly, allowing better visualization of the available evidence and gaps in scientific production. This process occurred through an inductive approach based on identifying patterns and a thematic analysis of the final set of information (27, 28).

## 3. Results

**Figure 1:**
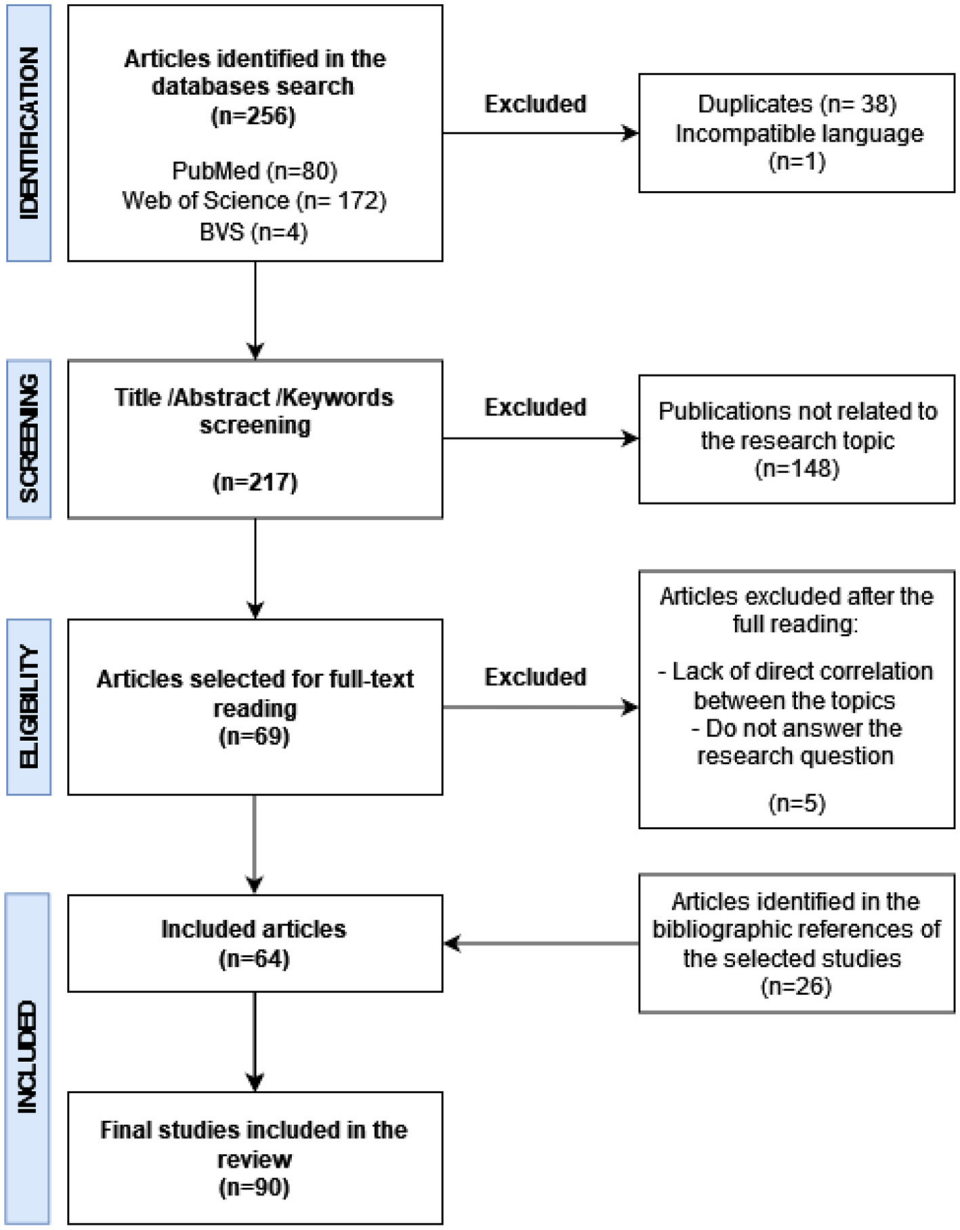
Flowchart summarizing the article selection process adopted in this review. Elaborated by the authors.

The database search initially identified 256 articles. Of this total, 172 were from the Web of Science, 80 were from PubMed, and only 4 were from the BVS. After the first screening, 38 duplicates and one article published in French were excluded. Another 148 articles were excluded after reading the title, abstract, and keywords. Thus, 69 articles were selected for a full reading. After this phase, 64 articles were included, along with another 26 studies collected from the references. Therefore, 90 articles were finally selected for this review. The selection process is summarized in Figure 1.

Most of the articles included were written in English [88], and only two were published in Portuguese. As for their origin, 38 studies were from the United States of America, 10 from the United Kingdom, 7 were from France, 6 were from Brazil, 4 were from Qatar, 4 were from Italy, 3 were from India, 2 from Ghana, 2 from Oman, and 2 were from Saudi Arabia; Bahrain, Belgium, the Democratic Republic of the Congo, Greece, Iran, Lebanon, Malaysia, the Netherlands, Nigeria, Tanzania, Turkey, and the United Arab Emirates contributed with one article each. Forty-two articles were published in 2020, 31 in 2021, and 17 in 2022.

Regarding methodology, most of the selected studies are case series (31 articles) and case reports (24 articles). Nevertheless, cohort studies (12 articles), case-control type studies (3 articles), cross-sectional studies (2 articles), and ecological studies (4 articles) were also present, as well as narrative reviews (6 articles), opinion articles/editorials (3 articles), systematic literature reviews (4 articles), and one meta-analysis. The selected articles were named A1 to A90, and their main characteristics are summarized in Table 3. An extended version of this table has been included in the Supplementary Table 1 and can be checked for more information about the sample features of each paper.

**Table 3:** Characterization of the 90 articles included in this integrative literature review, according to author/year of publication, origin country, type of study, level of evidence.

**ID**	**Author/Year**	**Country**	**Study type**	**Evidence level**
A1	Alghamdi and Kashari (29)	Saudi Arabia	Case report	VI
A2	Al-Hebshi et al. (30)	Saudi Arabia	Case series	VI
A3	Alkindi et al. (31)	Oman	Case control study	IV
A4	Al Yazidi et al. (32)	Oman	Cohort	IV
A5	Anusim et al. (33)	USA	Case series	VI
A6	Arlet et al. (34)	France	Case series	VI
A7	Azerad et al. (35)	Belgium	Case series	VI
A8	Balanchivadze et al. (36)	USA	Case series	VI
A9	Beerkens et al. (37)	USA	Case report	VI
A10	Boga et al. (38)	Turkey	Cross-sectional study	VI
A11	Chen-Goodspeed and Idowu (39)	USA	Case series	VI
A12	Clift et al. (40)	UK	Cohort	IV
A13	Cook (41)	UK	Opinion article	VII
A14	De Luna et al. (42)	France	Case series	VI
A15	Español et al. (43)	USA	Case report	VI
A16	Ershler and Holbrook (44)	USA	Case report	VI
A17	Freitas et al. (45)	Brazil	Case report	VI
A18	Fronza et al. (46)	Italy	Case report	VI
A19	Hall et al. (47)	UK	Ecological study	VI
A20	Hippisley-Cox et al. (48)	UK	Cohort	IV
A21	Hoogenboom et al. (49)	USA	Cohort	IV
A22	Hussain et al. (50)	USA	Case series	VI
A23	John and John (51)	India	Opinion article	VII
A24	John et al. (52)	India	Narrative review	VII
A25	Kasinathan et al. (53)	USA	Case report	VI
A26	Kehinde and Osundiji (14)	UK	Narrative review	VII
A27	Kingsley et al. (54)	Nigeria	Case report	VI
A28	Madany et al. (55)	USA	Case-control study	IV
A29	Mazloom et al. (56)	USA	Case series	VI
A30	McCloskey et al. (57)	UK	Case series	VI
A31	Merz et al. (58)	USA	Case-control study	IV
A32	Minniti et al. (59)	USA	Cohort	IV
A33	Mitchell et al. (60)	USA	Case series	VI
A34	Mucalo et al. (61)	USA	Ecological study	VI
A35	Noisette et al. (62)	USA	Case series	VI
A36	Noun et al. (63)	Lebanon	Narrative review	VII
A37	Nur et al. (64)	Netherlands	Case series	VI
A38	Okar et al. (65)	Qatar	Case report	VI
A39	Parodi et al. (66)	Italy	Case series	VI
A40	Sahu et al. (67)	USA	Case series	VI
A41	Sahu et al. (68)	USA	Narrative review	VII
A42	Sayad et al. (69)	Iran	Systematic review	V
A43	Sewaralthahab and Smith (70)	USA	Case series	VI
A44	Sheha et al. (71)	Egypt	Case report	VI
A45	Teulier et al. (72)	France	Case report	VI
A46	Dun et al. (73)	USA	Ecological study	VI
A47	De Luna et al. (74)	France	Case report	VI
A48	Subarna et al. (75)	UK	Case series	VI
A49	de Sanctis et al. (76)	Italy	Case series	VI
A50	Odièvre et al. (77)	France	Case report	VI
A51	Okar et al. (78)	Qatar	Case report	VI
A52	Panepintoa et al. (15)	USA	Case series	VI
A53	Heilbronner et al. (79)	France	Case series	VI
A54	Hardy et al. (80)	Ghana	Case series	VI
A55	Morrone et al. (81)	USA	Case series	VI
A56	Singh et al. (16)	USA	Cohort	IV
A57	Resurreccion et al. (82)	USA	Cohort	IV
A58	Telfer et al. (83)	UK	Ecological study	IV
A59	Appiah-Kubi et al. (84)	USA	Case series	VI
A60	Ramachandran et al. (85)	USA	Case series	VI
A61	Menapace and Thein (86)	USA	Opinion article (editorial)	VII
A62	AbdulRahman et al. (87)	Bahrein	Cross-sectional study	IV
A63	Sivalingam et al. (88)	UK	Narrative review	VII
A64	Jacob et al. (89)	USA	Case report	VI
A65	Elia et al. (90)	Brazil	Case series	VI
A66	Justino et al. (91)	Brazil	Case report	VI
A67	Quaresima et al. (82)	Italy	Case report	VI
A68	Allison et al. (92)	USA	Case report	VI
A69	Ali et al. (93)	Qatar	Case series	VI
A70	Attoh et al. (94)	Ghana	Case series	VI
A71	Campbell et al. (95)	USA	Cohort	IV
A72	Dejong et al. (96)	USA	Case report	VI
A73	Fuja et al. (97)	USA	Case report	VI
A74	Gupta et al. (98)	United Arab Emirates	Narrative review	VII
A75	De Jesus et al. (99)	Brazil	Integrative review	V
A76	Hippisley-Cox et al. (100)	UK	Cohort	IV
A77	Koh et al. (101)	USA	Case report	VI
A78	Lee et al. (102)	Malaysia	Meta-analysis	I
A79	Lubala et al. (103)	Democratic Republic of Congo	Case report	VI
A80	Mawalla et al. (104)	Tanzania	Case report	VI
A81	Mitra et al. (105)	USA	Case report	VI
A82	Santos et al. (106)	Brazil	Systematic review	V
A83	Silva-Pinto et al. (107)	Brazil	Case series	VI
A84	Singh et al. (108)	USA	Cohort	IV
A85	Tentolouris et al. (109)	Greece	Case series	VI
A86	Verma et al. (110)	USA	Cohort	IV
A87	Yurtsever et al. (111)	USA	Case series	VI
A88	Hoogenboom et al. (112)	USA	Systematic review	V
A89	Waghmare et al. (113)	India	Case series	VI
A90	Arlet et al. (114)	France	Cohort	IV

ID, Identification; USA, United States of America; UK, United Kingdom.

In the data extraction and analysis process, the articles were organized into six categories, as presented in Table 4: (1) sickle cell disease as a risk factor for unfavorable outcomes and/or atypical presentations of COVID-19; (2) sickle cell disease as a factor with no significant impact on COVID-19 outcomes; (3) sickle cell disease as a protective factor for the clinical progression of COVID-19; (4) COVID-19 as a trigger for sickle cell disease complications; (5) the role of sickle cell disease genotypes on the severity of COVID-19; and (6) therapeutic options. Category 1 encompasses 42 studies that, when considering parameters such as complications, hospitalization, need for mechanical ventilation, ICU admission, and death, suggest that individuals with sickle cell disease are at higher risk for worse outcomes in COVID-19 or demonstrate that the infection may present with unusual clinical patterns in this population. Fourteen articles are included in category 2; they gather empirical evidence supporting the idea that sickle cell disease has no crucial influence on the clinical course of COVID-19, and therefore the risk factors for those patients would be the same as those universally observed in the general population. As for the third category, 13 studies indicate that sickle cell disease may act as a protective feature against SAR-CoV-2 infection, either indirectly or biologically. Thirty-six articles pointing out that COVID-19 can trigger typical complications of sickle cell disease, such as vaso-occlusive crises and acute chest syndrome, are grouped in category 4. Category 5 comprises 24 papers that address the possible implications of the different sickle-cell disease-causing genotypes, as well as of the sickle-cell trait, on the clinical progression of SARS-CoV-2 infection. Finally, category 6 includes 22 studies that discuss the therapeutic management of COVID-19 in the sickle cell population, considering medical protocols and the use of certain drugs in these patients. Furthermore, it is worth noting that some articles are framed in more than one category.

**Table 4:** Organization of the included studies into categories.

**Category**	**Definition**	**Articles included**
Sickle cell disease as a risk factor for unfavorable outcomes and/or atypical presentations of COVID-19	It brings together studies supporting that individuals with sickle cell disease are at risk for developing worse outcomes in the setting of COVID-19, considering parameters such as complications, hospitalization, need for mechanical ventilation, ICU admission, and death. It also includes articles suggesting that SARS-CoV-2 infection may present in non-classical forms (atypical symptoms) in individuals with sickle cell disease.	A1, A10, A11, A12, A13, A15, A20, A23, A24, A25, A27, A34, A36, A38, A39, A41, A42, A45, A46, A49, A52, A56, A57, A58, A63, A65, A70, A71, A72, A73, A74, A75, A76, A78, A79, A80, A81, A82, A86, A87, A88, A89.
Sickle cell disease as a factor with no significant impact on COVID-19 outcomes	Encompasses articles advocating that sickle cell disease does not have a significant impact on the clinical course and/or outcomes of COVID-19, presenting empirical evidence that infection in this group follows the patterns observed in the general population. Thus, universally established risk factors, such as advanced age, presence of other comorbidities, and organ damage, would be the only predictors of a worse prognosis for COVID-19 among these individuals.	A3, A6, A14, A19, A21, A30, A31, A32, A43, A54, A60, A62, A64, A84.
Sickle cell disease as a protective factor for the clinical progression of COVID-19	It includes studies indicating that sickle cell disease may exert a protective effect toward the negative outcomes of COVID-19, either through indirect mechanisms, such as increased access to health services for this group and the influence of sickle cell disease-modifying drugs such as hydroxyurea; or through biological factors, related to the state of chronic inflammation, hemolytic anemia, and functional hyposplenism/asplenia.	A7, A8, A18, A22, A28, A29, A33, A35, A48, A55, A59, A66, A83.
COVID-19 as a trigger for sickle cell disease complications	Includes articles that consider the infection caused by SARS-CoV-2 as capable of triggering typical complications of sickle cell disease, such as vaso-occlusive crises and acute chest syndrome, conditions that are generally associated with high rates of morbidity and mortality.	A1, A2, A3, A4, A6, A9, A11, A15, A17, A22, A26, A27, A34, A36, A37, A38, A40, A41, A44, A45, A47, A50, A53, A54, A55, A59, A61, A64, A65, A66, A67, A68, A69, A80, A82, A85.
Role of sickle cell disease genotypes on the severity of COVID-19	It covers studies that explore the behavior of the different genotypes linked with sickle cell disease in COVID-19, including both articles that observe no differences in the clinical course of infection that might be associated with genotypes and those that suggest that milder or more severe genotypes are related to different outcomes in COVID-19. Studies that discuss the role of sickle cell trait in COVID-19 were also considered in this category.	A2, A3, A5, A6, A7, A8, A12, A13, A17, A21, A26 A32, A42, A43, A57, A58 A63, A65, A74, A86, A87, A88, A89, A90.
Therapeutic options	It addresses articles that explore the therapeutic possibilities for the management of COVID-19 specifically in individuals with sickle cell disease, such as tociluzumab, Voxelotor, ECMO, and blood transfusions, taking into consideration the pathophysiological particularities of sickle cell disease as well as the influences of clinical protocols and drugs usually recommended for this group.	A16, A29, A30, A38, A39, A45, A47, A50, A51, A53, A55, A59, A65, A68, A70, A71, A73, A77, A79, A82, A85, A88.

## 4. Discussion

The results reveal a decrease in scientific production on the subject between 2020 and 2022. The data also indicate that, although a reasonable number of publications met the inclusion criteria, most of them have a low level of evidence, i.e., they are mainly observational studies with reduced samples, retrospective data collection, and a lack of adequate control groups.

### 4.1. The impact of sickle cell disease on COVID-19: a risk factor, a protective factor, or a factor without significance?

Considering the pathophysiological aspects of sickle cell disease and the clinical observations made during the H1N1 epidemic, most experts prepared for a potential catastrophe regarding this group when the COVID-19 pandemic first became established. However, a fair number of case reports and case series have revealed surprisingly positive clinical courses and outcomes in this population (35, 36, 46, 50, 56, 60, 62, 75, 81, 84, 91).

In this regard, some authors argue that sickle cell disease may act as an indirect protective factor against severe forms of SARS-CoV-2 infection. As hypothesized by Sivalingam et al. (88), closer proximity to health care services and routines may have a beneficial influence by improving or speeding up access to medical monitoring and proper therapeutic regimens. In addition, organizations such as the CDC have recommended more meticulous clinical approaches for high-risk groups (13), and some countries have adopted protocols in this direction that included sickle cell patients. Thus, prior knowledge about the hematologic condition by health care professionals could also be associated with earlier diagnosis and the more aggressive administration of antivirals, anti-inflammatory drugs, anticoagulants, and blood transfusions, with a direct impact on the clinical course of COVID-19 (36, 56, 84).

Furthermore, the chronic use of sickle cell disease-modifying therapies, such as hydroxyurea, has been implicated as one of the possible causes of the positive outcomes of COVID-19 among sickle cell patients (59, 69, 75, 81, 90, 102, 111). By increasing fetal hemoglobin levels, hydroxyurea can reduce vaso-occlusive events (3, 115), but its role in viral infections is unclear. While some studies correlate continued use of the substance with better outcomes in COVID-19 (36, 59, 69, 75, 90) – Yurtsever et al. (111), for example, associate hydroxyurea use with a 17% reduction in the risk of ICU admission – others have found no significant difference between patients using or not using this drug (34, 61, 114). The mechanisms by which hydroxyurea possibly modulates the clinical course of COVID-19 are not fully elucidated, but it is postulated that the substance diminishes levels of inflammatory cytokines and lowers activation of neutrophils and endothelial adhesion molecules, also reducing the inflammatory cascade (59, 81). Due to its apparent anti-inflammatory and immunomodulatory effects, whether directly or indirectly (by fetal hemoglobin synthesis), some experts even argue that the drug can be repurposed to treat COVID-19 in people without sickle cell disease (116, 117), thereby supporting the hypothesis that it has protective effects on sickle cell patients.

Some authors argue that sickle cell disease may offer an actual biological advantage against SARS-CoV-2 infection. The characteristic state of chronic inflammation and hemolytic anemia associated with the condition would somehow protect individuals from severe presentations of COVID-19 (35, 46, 50, 91, 107), in a similar way to what occurs with sickle cell carriers in malaria. It is hypothesized that people with sickle cell disease have elevated levels of IFNα/β, which would contribute to the peculiar basal inflammatory activity in sickle cell status and could play a protective role regarding viral diseases, including SARS-CoV-2 infection (55). Chakravorty et al. (75), in contrast, suggests that hyposplenism would be able to inhibit the cytokine storm of COVID-19, preventing the severe outcomes of the infection.

Contrary to this, some authors advocate that sickle cell disease confers an increased risk for severe COVID-19 (41, 43, 51, 52, 54, 61, 63, 65, 66, 68, 69, 88, 94, 95, 98, 111, 112). More than one algorithm conceived to calculate the risk of different populations developing complications from COVID-19 pointed to sickle cell disease as a risk factor (48, 73, 88, 100) supporting, for example, the indication of the prophylactic use of anticoagulants for this group (53, 56, 65, 84). In fact, the hypercoagulability state typical of sickle cell disease and the capacity of COVID-19 to promote thromboembolic events are listed as reasons that could explain the possibly higher risk of adverse outcomes of the infection (52, 72, 99). Some studies suggest an elevated incidence of pulmonary embolism in people with sickle cell disease and COVID-19 (16, 43, 53, 72, 79, 103). However, case series, such as those conducted by Mitchell et al. (60) and Noisette et al. (62), present divergent data in this regard. Even more recently, in their cohort of 281 patients with sickle cell disease and COVID-19, Singh et al. (108) found no significant differences in rates of venous thromboembolism compared to the group of patients with sickle cell disease hospitalized for other reasons than SARS-CoV-2 infection. Therefore, we do not have clear evidence of the specific occurrences of thromboembolic complications among people with sickle cell disease in the context of COVID-19, and this is a topic that deserves future investigation.

Several studies describe severe presentations, increased rates of hospitalization, ICU admission, a need for mechanical ventilation, and mortality among people with sickle cell disease and COVID-19. Objectively, the hospitalization rate in this population ranged from 26 to 77.1%, the intubation rate was between 9 and 10%, and mortality varied from 3.2 to 8.4% (15, 16, 38, 39, 76, 83). Clift et al. (40) demonstrated a 2- to 4-fold increased risk of hospitalization for people with sickle cell disease and COVID-19 and an enhanced risk of death from the infection up to 2.6-fold in this population. Some reports have pointed out that the increased risks for poorer outcomes in this group remain even in the presence of novel variants of the virus, among vaccines, and among children, which are situations associated with less severe clinical presentations in the general public (95, 96, 118). In some cases, even individuals with sickle cell trait are considered to be at risk (40, 110, 112, 119). The immunosuppressive state, increased risk of sepsis, endothelial dysfunction, and cardiovascular impairment are some of the characteristics of sickle cell disease that may explain these outcomes (2, 51, 54, 61, 63, 66, 68). The fact that COVID-19 acts as a trigger for the occurrence of sickle cell complications, which are events that are associated with high mortality, may also contribute to this scenario, as will be discussed in the next section.

It has also been observed that COVID-19 may present atypically in this population, with hepatobiliary manifestations (29), hematological abnormalities (105, 111), and the absence of typical respiratory symptoms (33, 34, 64, 82). This picture could make it difficult to diagnose the infection and has led some authors to recommend testing individuals with sickle cell disease for COVID-19 even in the absence of classic symptoms (64, 68, 82). In the same spirit, some markers used to predict a worse prognosis in COVID-19, such as serum ferritin, which are often adopted for clinical evaluation in the general population, have a controversial application in this group. The frequent blood transfusions and the state of chronic inflammation lead to higher baseline levels of ferritin in individuals with sickle cell disease. Thus, this parameter does not appear to have good predictive value for identifying immune dysregulation associated with SARS-CoV-2 infection in these individuals, which should also be considered by health professionals in their routine (102).

Finally, some studies suggest that when it comes to COVID-19, individuals with sickle cell disease or sickle cell trait have similar rates of death, hospitalization, and ICU admission compared to people without sickle cell disease or sickle cell trait (31, 34, 42, 47, 49, 58, 70, 80, 85, 87, 108). These papers did not identify mechanisms related to sickle cell disease that act directly in the clinical course of COVID-19 but instead highlighted features also observed in the general population, such as the existence of other comorbidities, advanced age, and the presence of organ damage, as risk factors for unfavorable outcomes (57, 59).

### 4.2. COVID-19 as a trigger for sickle cell disease complications

Similar to what is observed for other viral infections, COVID-19 appears to be able to precipitate acute complications typical of sickle cell disease. These manifestations have been reported since the beginning of the pandemic with significant frequency, although the cause-effect correlation cannot always be clearly determined. There are points of disagreement about the biological impact of COVID-19 in individuals with sickle cell disease, but most authors seem to agree that SARS-CoV-2 can act as a trigger for vaso-occlusive crises and acute chest syndrome (14, 29, 30, 32, 34, 37, 39, 42, 43, 45, 46, 67, 71, 72, 74, 75, 77, 79, 82, 84, 86, 90, 92, 98, 104, 106, 109, 112, 118). Explanations for this are grounded in empirical data and well-established knowledge about the mechanisms by which infections trigger complications of sickle cell disease. During an infectious process, there is an exacerbation of the chronic inflammatory state inherent in sickle cell disease. The recruitment of leukocytes, the release of cytokines, and direct injury to the endothelium further increase the hypercoagulability phenomena, with amplification of vasoconstriction, platelet stimulation, and secretion of adhesion molecules, propitiating the occurrence of vaso-occlusion and associated symptoms (2, 3).

There is not much evidence on the real impact of these events on the morbidity and mortality of COVID-19 among individuals with sickle cell disease, but acute chest syndrome is one of the leading causes of death in this population (6). Therefore, there is a substantial potential for an unfavorable clinical course in these cases. In addition, sickle cell complications are associated with higher rates of hospitalization (72, 88) and, of course, with the expenditure of more therapeutic resources, such as oxygen supplementation, antibiotic therapy, and blood transfusions. In contrast, Alkindi et al. (31) show that the impact of COVID-19 on the morbidity and mortality of vaso-occlusive crises among individuals with sickle cell anemia was not significant. The occurrence of other sickle cell complications in the context of COVID-19, such as osteonecrosis, acute hemolytic crisis, post-transfusion hyperhemolysis syndrome, and splenic sequestration, has also been reported, although in a minor incidence (31, 65, 89, 97).

### 4.3. The influence of different genotypes

Some studies suggest that the clinical course of SARS-CoV-2 infection in sickle cell disease patients may vary according to their genotype. In certain samples, more severe cases of COVID-19 were seen in milder genotypes, especially the HbSC genotype, while among genotypes associated with more severe manifestations of sickle cell disease, such as HbSS and HbSβ-, COVID-19 was more commonly observed in its milder form (34, 35, 83, 88, 90, 114). At first glance, these observations are consistent with the hypotheses that chronic inflammation and hemolytic anemia could exert favorable effects on COVID-19 since these mechanisms would be more “active” in individuals with more severe genotypes. It is also worth noting that sickle cell individuals with severe genotypes are those who most often use hydroxyurea (59, 112), which could also have a favorable influence on the clinical course of COVID-19, as discussed earlier. In the Arlet et al. (114) cohort, the HbSC genotype was associated with a higher risk of mortality and development of thromboembolic events compared to the other groups (SS/Sβ-/Sβ+). According to these authors, this finding could be explained by SC hemoglobinopathy-specific endothelial dysfunctions; this would also explain higher mortality rates associated with this genetic profile in dengue cases, for example. There is, however, disagreement on the subject. Other studies, with similar levels of evidence, show more severe cases and higher hospitalization rates in individuals with HbSS and HbSSβ- genotypes (31, 36) or conclude that there is no relationship between sickle cell disease genotypes and the severity of COVID-19 (33, 59, 69).

The role of sickle cell trait in the clinical course of COVID-19 was also evaluated. Some researchers believed that the higher mortality observed among individuals from ethnic groups of African origin in the United Kingdom and the United States could be explained by differences in hemoglobin-producing genes, given the high prevalence of a sickle cell trait in this population (14, 41).

Resurreccion et al. (119) note that, even to a minor extent, carriers of the trait exhibit an increased mortality trend, as well as having a significantly higher cumulative risk if they have other associated comorbidities, such as diabetes. In addition, the study published by Verma et al. (110) reported that people with sickle cell trait are more likely to suffer from chronic kidney disease, diabetic kidney disease, hypertensive kidney disease, pulmonary embolisms, and cerebrovascular illnesses. Ultimately, this combination of factors appears to increase the risk of these individuals having worse outcomes in COVID-19, and independently, the infection itself seems to raise the risk for these individuals to develop acute kidney failure. Another paper reports a higher rate of intensive care unit admission in this population, as high as 36.4% (70). Furthermore, Clift et al. (40) reveal a 1.38 increased risk of hospitalization and a 1.51 increased risk for death among individuals with sickle cell trait and COVID-19.

At least two case reports describe individuals who discovered the status of sickle cell trait carrier at the onset of COVID-19, which triggered vaso-occlusive symptoms and endothelial dysfunction for the first time, making the diagnosis feasible. These situations also reveal a possible relationship of greater severity between the two clinical entities (45, 71).

The influence of the sickle cell trait on the evolution of COVID-19 remains controversial. Most papers refute the idea of increased risk, arguing that, after adjusting for the variables, sickle cell carriers have no significant differences in SARS-CoV-2 mortality compared with those without the trait. Socioeconomic vulnerabilities, from this point of view, appear to better explain the health disparities observed among the black population in the United Kingdom and the United States (15, 30, 49, 58, 69).

### 4.4. Therapeutic options

Blood transfusions, especially exchange transfusions, may benefit individuals with sickle cell disease and COVID-19, especially in the presence of significant anemia and acute chest syndrome (56, 57, 65, 74, 78, 81, 84, 90, 92, 106). However, there are disagreements regarding the early or prophylactic use of this measure. While some authors suggest that transfusion may be considered as early as possible (56, 65, 72, 78, 84, 90, 92), others believe that the criteria for use should be individualized and based on existing indication protocols (81). Likewise, there is no consensus when it comes to prophylactic anticoagulation. Many authors consider it plausible to intensify these regimens among sickle cell patients, given the potentially increased risk for coagulation disorders in this group (94, 103). Others, such as Singh et al. (108), facing the evidence that the risks are similar to those observed in the general population, suggest that anticoagulation should be carried out rationally, following the guidelines adopted in each institution.

A case report discusses the use of Voxelotor, an inhibitor of HbS polymerization that increases the affinity of hemoglobin for oxygen, as an alternative for sickle cell disease patients infected with SARS-CoV-2 to avoid transfusions (44). Approved by the Food and Drug Administration in 2019, this substance has been used in the treatment of sickle cell disease, usually in continuous schemes (93). The application in an acute situation, in an individual with pneumonia, falling hemoglobin levels, and a history of transfusion of two units of leukoreduced red blood cells without response, was quite satisfactory, leading to improvements in clinical and laboratory parameters that were observed after the second day of administration, without the need to resort to erythrocytapheresis (44).

The administration of tocilizumab, an anti-human IL-6 receptor monoclonal antibody that inhibits signal transduction by binding to sIL-6R and mIL-6R, has been described in some case reports of sickle cell individuals infected by the SARS-CoV-2, demonstrating successful results (74, 77, 79, 97). This resource, which has even been made available to the general population in some places, should be considered for patients at higher risk of developing a hyperinflammatory syndrome or sickle cell complications, for example (74, 120).

It was identified that four cases of extracorporeal membrane oxygenation (ECMO) had been performed in patients with sickle cell disease infected with SARS-CoV-2, all of which had relatively satisfactory outcomes (66, 72, 101). The use in patients with acute chest syndrome is particularly noteworthy, considering that ECMO has unfavorable repercussions and is not usually indicated in these situations. The successful application in these cases may indicate that the pathophysiology of this sickle cell complication in the setting of COVID-19 has particularities when contrasting with other triggering viral conditions (72, 101).

It should also be emphasized that oxygen support, antibiotic therapy, analgesia, and hydration—pillars in the management of acute episodes of sickle cell disease—must not be ignored or underestimated in COVID-19, even to the detriment of more modern therapeutic options. It is crucial to use these measures as soon as possible to ensure patient stabilization and reduce morbidity and mortality (72).

Vaccination, an essential tool for reducing mortality from infectious causes in this population, should also be encouraged in all age groups, as it has been for the general population (2, 47, 106). Studies show that there may be some hesitation among people with sickle cell disease regarding the safety profile of immunizers against SARS-CoV-2 and the occurrence of adverse effects (121). Therefore, professionals must clarify possible myths and highlight the proven benefits of vaccination. Studies, such as Friedman et al. (122), which show no association between vaccination and the occurrence of pain episodes in this population, can be used to corroborate the safety of the COVID-19 vaccines.

### 4.5. Study limitations

Despite the methodological rigor, this study has some limitations. One of them is temporal, as we included articles published up to October 2022. Although there is a downward trend in scientific production as the pandemic advances, it is possible that relevant studies published later were missed.

Another limitation is regarding the lower levels of evidence of most of the primary articles included in the review, which prevents us from postulating more robust generalizations and indicates the need to expand the research on the subject. In addition, considering that this study included articles from several countries, it is likely that varying treatment guidelines for COVID-19 across these regions and over the period considered in the search make it difficult to generalize the findings about this specific topic.

## 5. Conclusion

Data analysis available in the literature on the impact of COVID-19 in individuals with sickle cell disease reveals many divergences, especially about the role of molecular mechanisms associated with sickle cell pathogenesis, such as chronic inflammation, hypercoagulability, and hemolytic anemia; and factors of possible indirect influence, namely the use of hydroxyurea and access to medical care. The studies lead us to conclude, however, that SARS-CoV-2, similar to other viral agents, is capable of acting as a precipitator of typical sickle cell disease events, especially acute chest syndrome and vaso-occlusive crises, historically linked to increased morbidity and mortality. Moreover, COVID-19 has the potential to present clinically in an atypical form in this group, often with the absence of respiratory symptoms.

Thus, more than outlining individuals with sickle cell disease as part or not of a risk group, this information highlights the need to direct investments toward robust research that is able to broaden the understanding of the particularities of the clinical course of SARS-CoV-2 infection in the context of sickle cell disease, including the expansion of topics such as incidence of thromboembolic events and the role of different genotypes. Providing insight into these fields may also contribute to a better understanding of the underlying pathophysiology of sickle cell disease and COVID-19. In addition, the data draw attention to the importance of capacitating healthcare services and professionals regarding the management of SARS-CoV-2-infected sickle cell individuals. Specific guidelines and therapeutic protocols should include close monitoring, extensive testing, and the use of different therapeutic resources according to the severity of the clinical presentation. Public policies directed to sickle cell individuals must be considered.

## Data availability statement

The original contributions presented in the study are included in the article/Supplementary material, further inquiries can be directed to the corresponding author.

## Author contributions

LP: conceptualization, data curation, formal analysis, interpretation, and drafting the original manuscript. MS: data curation, formal analysis, interpretation, and reviewing the manuscript. CG and IE: formal analysis, interpretation, and reviewing the manuscript. DM: conceptualization, data curation, formal analysis, interpretation, project management, and reviewing the manuscript. All authors participated in the final approval of the version submitted.
