# Supplementary material for: Impact of the SARS-CoV-2 infection in individuals with sickle cell disease: an integrative review
Source: Front Med (Lausanne). 2023 May 2;10:1144226. doi: 10.3389/fmed.2023.1144226 (PMC10187638; doi:10.3389/fmed.2023.1144226)
Supplement: Supplementary file 1 [file Table_1.DOCX]

Supplementary Material

Impact of the SARS-CoV-2 infection in individuals with sickle cell disease: an integrative review

Laura Resende Guimarães Pereira, Maria Vitoria Gomes da Silva, Carla Maria Ramos Germano, Isabeth da Fonseca Estevão^,^ Débora Gusmão Melo^*^

*** Correspondence:** Débora Gusmão Melo: [dgmelo@ufscar.br](mailto:dgmelo@ufscar.br)

**Form 1** - **Eligibility form of the studies identified in the searched databases.**

**ARTICLE IDENTIFICATION**

Title:

Authorship:

Journal in which it was published:

Year of publication: Volume: First-page number:

**ELIGIBILITY CRITERIA**

1. TYPE OF STUDY:

2. LANGUAGE OF THE STUDY

( ) Portuguese ( ) English ( ) Spanish

3. PARTICIPANTS

Does it involve discussions about patients diagnosed with sickle cell disease?

( ) Yes ( ) No ( ) Unclear

4. INTEREST

Does it address SARS-CoV-2 infection and pandemic in the context of sickle cell disease?

( ) Yes ( ) No ( ) Unclear

5. CONCLUSIONS:

**FINAL DECISION**

( ) Include ( ) Exclude ( ) Unclear

**Form 2 - Instrument for extracting data from the selected studies.**

**IDENTIFICATION OF THE STUDY:**

| Bibliographic reference of the selected article: |
| --- |
| Study location and period of execution: |

**METHODOLOGICAL FEATURES OF THE STUDY:**

| Type of publication | Methods (detailed) |
| --- | --- |
| Objectives of the study | Inclusion and exclusion criteria |
| Participants' characteristics/ Sample features | Data analysis |

**RESULTS OF THE STUDY:**

| Outcomes: |
| --- |
| Results (given the objectives of this review, what are the main results of the study analyzed?): |
| Study limitations: |
| Level of evidence: |

# Supplementary Table 1. Characterization of the 90 articles included in this integrative literature review, according to author/year of publication, origin country, type of study, level of evidence, and sample features.

| ID | Author/Year | Country | Study type | Evidence level | Sample characteristics |
| --- | --- | --- | --- | --- | --- |
| A1 | Alghamdi & Kashari, 2020 | Saudi Arabia | Case report | VI | 1 patient (5 years old, genotype HbSβ+) |
| A2 | Al-Hebshi et al., 2020 | Saudi Arabia | Case series | VI | 3 patients (14, 12, and 50 years old; HbSS and SCT genotypes). |
| A3 | Alkindi et al., 2021. | Oman | Case control study | IV | 100 patients with SCD and VOC (50 in the COVID group and 50 in the non-COVID group). |
| A4 | Al Yazidi et al., 2021. | Oman | Cohort | IV | 56 patients (all under 14 years old) |
| A5 | Anusim et al., 2021. | USA | Case series | VI | 11 patients [genotypes HbSS (5); HbSC (4); HbS/β+ (1) and HbS/α (1)] |
| A6 | Arlet et al., 2020. | France | Case series | VI | 83 patients (ages 3 to 68 years, diverse genotypes) |
| A7 | Azerad et al., 2020. | Belgium | Case series | VI | 3 patients (HbSS genotypes, ages 23-44). |
| A8 | Balanchivadze et al., 2020. | USA. | Case series | VI | 24 patients (18 had SCT, 4 had HbSS genotype, 1 had HbS/β+ and one had HbSC). |
| A9 | Beerkens et al., 2020. | USA | Case report | VI | 1 patient (genotype HbSβ°, 21 years, male). |
| A10 | Boğa et al., 2021. | Turkey | Cross-sectional study | VI | 39 adults with SCD (mostly with HbSS genotype) and 121 health professionals, ages 18 to 50 years, without comorbidities. |
| A11 | Chen-Goodspeed & Idowu, 2021. | USA | Case series | VI | 5 patients (aged 23-49 years, with HbSS and HbSC genotypes). |
| A12 | Clift et al., 2021. | UK | Cohort | IV | 5,059 (0.04%) participants were patients with SDC and 25,682 (0.21%) were patients with SCT. |
| A13 | Cook, 2021. | UK | Opinion article | VII | - |
| A14 | De Luna et al., 2020. | France | Case series | VI | 305 patients with SCD. |
| A15 | Español et al., 2022. | USA | Case report | VI | 1 patient (8 years old, female, HbSS genotype). |
| A16 | Ershler & Holbrook, 2020. | USA | Case report | VI | 1 patient (female, 39 years old, HbSS genotype). |
| A17 | Freitas et al., 2021. | Brazil | Case report | VI | 1 patient (male, 63 years old, SCT discovered as a result of COVID-19, history of HBP). |
| A18 | Fronza et al., 2020. | Italy. | Case report | VI | 1 patient (female, 44 years old, Senegalese, history of SCD). |
| A19 | Hall et al., 2021. | UK. | Ecological study | VI | 256 adult patients (57% had HbSS genotype and 43% had heterozygous genotypes- HbSC/HbSb+/others). |
| A20 | Julia Hippisley-Cox et al., 2021. | UK | Cohort | IV | 6,952,440 patients were enrolled in the study, of which 2073 (0.03%) had SCD. |
| A21 | Hoogenboom et al., 2021. | USA | Cohort | IV | 12,659 patients; 53 had SCD (74% HbSS, 21% HbSC and 6% HbS/b) and 62 had SCT. |
| A22 | Hussain et al., 2020. | USA | Case series | VI | 4 patients (22 to 41 years, HbSS and HbSC genotypes). |
| A23 | John & John, 2020. | India | Opinion article | VII | - |
| A24 | John et al., 2021. | India | Narrative review | VII | - |
| A25 | Kasinathan et al., 2020. | USA | Case report | VI | 1 patient (female, 20 years old, genotype HbSC). |
| A26 | Kehinde & Osundiji, 2020. | UK. | Narrative review | VII | 15 case reports of patients with SCD and COVID-19 published in the literature were analyzed. |
| A27 | Kingsley et al., 2021. | Nigeria | Case report | VI | 1 patient (male, 42 years old) with SCD. |
| A28 | Madany et al., 2021. | USA | Case-control study | IV | 24 SCD patients. |
| A29 | Mazloom et al., 2020. | USA | Case series | VI | 13 patients (mean age 34 years, 7 patients had HbSS genotype and 6 had HbSC genotype). |
| A30 | McCloskey et al., 2020. | UK | Case series | VI | 10 patients, most with HbSS genotype, ages ranging from 23 to 57 years. |
| A31 | Merz et al., 2021. | USA | Case-control study | IV | 166 African-American patients who tested positive for SARS-CoV-2, of which 20 were SCT carries and 3 were hemoglobin C trait carries. |
| A32 | Minniti et al., 2021. | USA | Cohort | IV | 66 patients with SCD and COVID-19, ages 8 months to 69 years, most had HbSS genotype. |
| A33 | Mitchell et al., 2020. | USA | Case series | VI | 54 patients with COVID-19, ages 2 months to 30 years, 12 of these patients had sickle cell anemia. |
| A34 | Mucalo et al., 2021. | USA | Ecological study | VI | 750 patients with SCD and COVID-19 (ages 0-40 years, various genotypes). |
| A35 | Noisette et al., 2020. | USA | Case series | VI | 23 patients with SCD and COVID-19; 19 had HbSS genotype, 2 had HbSC and 2 were HbSβ+. |
| A36 | Noun et al., 2020. | Lebanon | Narrative review | VII | Nine articles reporting cases of COVID-19 in individuals with SCD were selected. In total, 116 patients were evaluated. |
| A37 | Nur et al., 2020. | Netherlands | Case series | VI | 2 patients (1 male and 1 female, both with HbSC genotype and ages 24 and 20 years). |
| A38 | Okar et al., 2021. | Qatar | Case report | VI | 1 patient (male, 48 years old, with SCD). |
| A39 | Parodi et al., 2021. | Italy | Case series | VI | 2 pediatric patients (9 and 20 months, one female and one male with no previous diagnosis of SCD). |
| A40 | Sahu et al., 2021. | USA | Case series | VI | 5 patients with DF and COVID-19, aged 21 to 47 years; 2 had HbSS genotype, 2 had HbSC, and 1 had HbS/β° |
| A41 | Sahu et al., 2020. | USA | Narrative review | VII | Six studies were selected, amounting 19 patients with SCD and COVID-19 (confirmed by RT-PCR or suggestive clinical data). |
| A42 | Sayad et al., 2021. | Iran | Systematic review | V | 27 published articles were included, as well as data from the international and American registries of SCD, which at the time included 134 adult patients and 44 patients under the age of 19. |
| A43 | Sewaralthahab & Smith, 2020. | USA | Case series | VI | 61 African American patients with SCD or SCT (21 were hospitalized - 9 had SCD- genotypes HbSS, HbSC, HbSβ; 11 patients were SCT carries) and 40 were seen on an outpatient basis only. |
| A44 | Sheha et al., 2020. | Egypt | Case report | VI | 1 patient, female, 22 years old, with a previously unknown diagnosis of sickle cell disease/trait. |
| A45 | Teulier et al., 2021. | France | Case report | VI | 1 patient, male, 33 years old, HbSS genotype. |
| A46 | Dun et al., 2020. | USA | Ecological study | VI | 534,023 patients with COVID-19, age 65 years or older. Of this total, 222 individuals with SCD were identified. |
| A47 | De Luna, Habibi, et al., 2020. | France | Case report | VI | 1 patient, male, 45 years old, genotype HbSC. |
| A48 | Subarna Chakravorty et al., 2020. | UK | Case series | VI | 10 patients (all had HbSS genotype, ages ranging from 25 to 54 years). |
| A49 | de Sanctis et al., 2020. | Italy. | Case series | VI | 13 patients with COVID-19 and previously diagnosed with hemoglobinopathies, 3 of them had SCD. |
| A50 | Odièvre et al., 2020. | France | Case report | VI | 1 patient, female, 16 years old, HbSS genotype. |
| A51 | Okar, Aldeeb, et al., 2021. | Qatar | Case report | VI | 1 patient; male, 22 years old, with SCD. |
| A52 | Panepintoa et al., 2020. | USA | Case series | VI | 178 patients with COVID-19 and SCD, the mean age was 28.6 years and 76% of the patients had HbSS or HbSβ° genotype. |
| A53 | Heilbronner et al., 2020. | France | Case series | VI | 12 patients with SCD, ages 5 and 17.5 years, only 4 tested positive for COVID-19, all had HbSS genotype. |
| A54 | Hardy et al., 2021. | Ghana | Case series | VI | 3 patients with confirmed COVID-19, all female, aged 20-34 years, genotypes HbSC (2) and HbSS (1). |
| A55 | Morrone et al., 2020. | USA | Case series | VI | 8 SCD patients with age ≤ 22 years, who tested positive for SARS-CoV-2 and met the criteria for ACS. Most of them (8) had HbSS genotype. |
| A56 | Singh et al., 2021. | USA | Cohort | IV | 312 patients with COVID-19 and SCD and 449 patients with COVID-19 and SCT. The control group included 45,517 black patients without SCD/SCT and with COVID-19. |
| A57 | Resurreccion et al., 2021. | USA | Cohort | IV | 729 patients with SCT who were identified in UK Biobank, including 14 people with SCT who also had a diagnosis of COVID-19. |
| A58 | Telfer et al., 2020. | UK | Ecological study | IV | 195 patients with hemoglobinopathies and confirmed or suspected COVID-19; 166 of the cases were from people with SCD, 129 of them had severe genotypes (HbSS or HbSβ0-) and 37 mild genotypes (HbSC, HbSβ+- or HbSE). Most were adults and female. |
| A59 | Appiah-Kubi et al., 2020. | USA | Case series | VI | 7 patients, ages 2 to 20 years, most were female (5/7) and had HbSS genotype (6/7). |
| A60 | Ramachandran et al., 2020. | USA | Case series | VI | 9 patients with COVID-19 and SCD, most of them had HbSS genotype, except for 1 who had HbSC genotype. |
| A61 | Menapace & Thein, 2020. | USA | Opinion article (editorial) | VII | - |
| A62 | AbdulRahman et al., 2020. | Bahrein | Cross-sectional study | IV | 38,092 patients were tested for COVID-19; 378 (1%) had SCD. Six of these patients with SCD were diagnosed with COVID-19 (1.6%). |
| A63 | Sivalingam et al., 2020. | UK | Narrative review | VII | - |
| A64 | Jacob et al., 2020. | USA | Case report | VI | 1 patient, male, 2 years and 9 months old, with SCD, HbSS genotype. |
| A65 | Elia et al., 2021. | Brazil | Case series | VI | 3 pediatric patients, ages 7 (M), 10 (F) and 16 (F) years, the first one had HbSC genotype and the other two had HbSS genotype. |
| A66 | Justino et al., 2020. | Brazil | Case report | VI | 1 patient, female, 35 years old, with SCD, at the 28th week of pregnancy |
| A67 | Quaresima et al., 2020. | Italy | Case report | VI | 1 patient, female, 18 years old, HbSS genotype and rare blood type. |
| A68 | Allison et al., 2020. | USA | Case report | VI | 1 patient, male, 27 years old, HbSC genotype. |
| A69 | Ali et al., 2022. | Qatar | Case series | VI | 2 patients, both female, ages 39 and 52, known history of SCD (one with unknown genotype and the other with HbSS genotype). |
| A70 | Attoh et al., 2022. | Ghana | Case series | VI | 3 patients, two males with genotype HbSS and one female with genotype HbSC. Ages 12 (M), 32 (F) and 59 years (M). |
| A71 | Campbell et al., 2022. | USA | Cohort | IV | 1877 patients with COVID-19 were included in the primary analysis. The mean age was 15.3 years and 53% of the sample was female. Of this total, 27 adolescents had SCD. |
| A72 | Dejong et al., 2022. | USA | Case report | VI | 1 patient, female, 14 years old, HbSS genotype. |
| A73 | Fuja et al., 2022. | USA | Case report | VI | 1 patient, male, adult, HbSS genotype. |
| A74 | Gupta et al., 2022. | United Arab Emirates | Narrative review | VII | - |
| A75 | De Jesus et al., 2021. | Brazil | Integrative review | V | 15 articles were selected in this review. |
| A76 | J Hippisley-Cox et al., 2022. | UK | Cohort | IV | 1.3 million patients with COVID-19 were considered in the derivation cohort and 0.15 million people were included the validation cohort, ages 18 to 100 years. In the derivation cohort, 2682 patients had SCD/HIV or other immunodeficiencies. In the validation cohort, this group contained 383 individuals. |
| A77 | Koh et al., 2022. | USA | Case report | VI | 1 patient, male, 11 years old, genotype HbSS. |
| A78 | Lee et al., 2022. | Malaysia | Meta-analysis | I | 11 studies were selected; 352 patients were analyzed in total. In the meta-analysis, only 10 studies were considered. |
| A79 | Lubala et al., 2022. | Democratic Republic of Congo | Case report | VI | 1 patient, male, 3 months old, HbSS genotype. |
| A80 | Mawalla et al., 2022. | Tanzania. | Case report | VI | 1 patient, male, 30 years old, HbSS genotype. |
| A81 | Mitra et al., 2022. | USA | Case report | VI | 1 patient, female, 31 years old, genotype HbSS, pregnant (32nd week). |
| A82 | Santos et al., 2022. | Brazil | Systematic review | V | 7 studies were selected. |
| A83 | Silva-Pinto et al., 2022. | Brazil | Case series | VI | 10 patients (9 adults, ages 28-45 years, and 1 child, 8 years), most of them were female (8/10) and had HbSS genotype (6/10). |
| A84 | Singh et al., 2022. | USA | Cohort | IV | 281 patients with SCD and COVID-19 in the group of interest and 4,873 patients with SCD and without COVID-19 diagnosis in the control group. |
| A85 | Tentolouris et al., 2022. | Greece | Case series | VI | 3 patients, two males and one female, ages 45, 51, 54 years, all of them had HbS/β genotype. |
| A86 | Verma et al., 2022. | USA | Cohort | IV | 132,577 patients tested for COVID-19 were considered; 2,729 were SCT carriers and 129,848 were non-SCT carriers. |
| A87 | Yurtsever et al., 2021. | USA | Case series | VI | 40 patients (27 adults and 13 children), with HbSS, HbSC, HbSβ-e and HbSβ+ genotypes. |
| A88 | Hoogenboom et al., 2022. | USA | Systematic review | V | 71 articles were selected. Overall, 2,290 patients with SCD and 1,937 patients with SCT were analyzed. |
| A89 | Waghmare et al., 2022. | India | Case series | VI | 31 patients, pregnant and postpartum women,  6 of them had HbSS, 1 had HbSβ genotype, and 24 had SCT. |
| A90 | Arlet et al., 2022. | France | Cohort | IV | 319 patients with SCD, mean age 27.4, 50.5% were male and 27% were aged < 18 years; 216 (86.5%) had the HbSS or HbSβ- genotype and 33 (10.3%) had HbSC genotype. |

Abbreviations: ID= identification; SCD= sickle cell disease; SCT= sickle cell trait; VOC= vaso-occlusive crisis; ACS= acute chest syndrome; USA= United States of America; UK= United Kingdom; HBP= high blood pressure; M= male, F=female.
